# Multimodal label-free imaging of living dermal equivalents including dermal papilla cells

**DOI:** 10.1186/s13287-018-0838-9

**Published:** 2018-04-03

**Authors:** Aleksandra V. Meleshina, Olga S. Rogovaya, Varvara V. Dudenkova, Marina A. Sirotkina, Maria M. Lukina, Alena S. Bystrova, Victoria G. Krut, Daria S. Kuznetsova, Ekaterina P. Kalabusheva, Andrey V. Vasiliev, Ekaterina A. Vorotelyak, Elena V. Zagaynova

**Affiliations:** 1 Institute of Biomedical Technologies, Nizhny Novgorod State Medical Academy, Minin and Pozharsky Square, 10/1, Nizhny Novgorod, 603005 Russia; 2 Institute of Biology and Biomedicine, Nizhny Novgorod State University, Gagarin Avenue, 23, Nizhny Novgorod, 603950 Russia; 3 Department of Radiophysics, Nizhny Novgorod State University, Gagarin Avenue, 23, Nizhny Novgorod, 603950 Russia; 4 Koltzov Institute of Developmental Biology, Russian Academy of Sciences, Moscow, 119991 Russia; 5 Department of Regenerative Medicine, Research Institute of Translational Medicine, Pirogov Russian National Research Medical University, Moscow, 117997 Russia; 6 Department of Cell Biology and Histology, Faculty of Biology, Moscow State University, Moscow, 119991 Russia

**Keywords:** Dermal equivalents, Dermal papilla cells, NAD(P)H, CP OCT, MPT, FLIM

## Abstract

**Background:**

Despite the significant progress in the development of skin equivalents (SEs), the problem of noninvasively assessing the quality of the cell components and the collagen structure of living SEs both before and after transplantation remains. Undoubted preference is given to in vivo methods of noninvasive, label-free monitoring of the state of the SEs. Optical bioimaging methods, such as cross-polarization optical coherence tomography (CP OCT), multiphoton tomography (MPT), and fluorescence lifetime imaging microscopy (FLIM), present particular advantages for the visualization of such SEs.

**Methods:**

In this study, we simultaneously applied several visualization techniques for skin model examination. We investigated the structure and quality of dermal equivalents containing dermal papilla (DP) cells and dermal fibroblasts (FBs) using CP OCT, MPT, and FLIM. Both the energy metabolism of the cell components and the structuring of the collagen fibrils were addressed.

**Results:**

Based on the data from the fluorescence lifetimes and the contributions of protein-bound NAD(P)H, a bias toward oxidative metabolism was indicated, for the first time, in both the DP cells and FBs on day 14 of SE cultivation. The CP OCT and MPT data also indicated that both DP cells and FBs structured the collagen gel in a similar manner.

**Conclusion:**

In this study, multimodal label-free imaging of the structure and quality of living dermal equivalents was implemented for the first time with the use CP OCT, MPT, and FLIM of NAD(P)H. Our data suggest that the combination of different imaging techniques provides an integrated approach to data acquisition regarding the structure and quality of dermal equivalents, minimizes the potential disadvantages of using a single method, and provides an ideal information profile for clinical and research applications.

## Background

The use of tissue engineering for the treatment of acute and chronic wounds, diabetic ulcers, and burns is an important and rapidly developing area of modern regenerative medicine [1, 2]. The main directions of such developments are in the isolation and cultivation of cell cultures *in vitro,* with subsequent tissue reconstruction, the study of stem cell properties, the role of the microenvironment, and the study of biocompatible synthetic materials.

The result of work in these areas is the creation of histotypical or functional analogs (equivalents) of tissues and organs, in particular human skin equivalents. Skin equivalents (SEs) are already used clinically to promote the healing of acute and chronic wounds or in pharmaceutical research as test systems [3].

Human SEs are bioengineered structures (skin substitutes) consisting of cell components, i.e., cultured human skin cells and a substrate (matrix scaffold; an analog of the intracellular matrix) [4]. For the vast majority of research on wound healing it is fibroblasts and/or keratinocytes that have been used as the source of the cell components. However, tissue-engineering structures using stem cells (SCs) have been developed [5] over the same period. Such development of methods for treating injuries and wound healing involves mainly adult SCs, particularly multipotent mesenchymal stromal cells (MSCs) [6].

Despite the significant progress in developing SEs, a problem remains for noninvasively assessing the quality of the cell components and the collagen structure of the living SEs both before and after transplantation. For this, various biological and medical approaches have been used (for example, histology and immunohistochemistry) [7, 8]. However, undoubted preference is given to *in vivo* methods of noninvasive, label-free monitoring of the state of the SEs. Optical bioimaging methods, such as optical coherence tomography (OCT) and multiphoton imaging that are already recognized for providing particular advantages to the solution of other diagnostic problems in clinical practice, can also be used for visualization of the skin equivalents [9].

OCT is a method of *in vivo* monitoring of biological tissue structures to depths of up to 1.5 mm, with a resolution of 10–15 μm [10]. Recently the possibilities of using traditional OCT have been substantially expanded by the addition of a polarization-sensitive mode [11] and by microangiography [12]. Cross-polarization OCT (CP OCT) reflects the polarization properties of biological tissues and provides information about the presence of organized structures—the concentration and type of collagen fibers and their local orientation in the surface layers. The method of CP OCT, in comparison with “traditional” OCT, is based on obtaining images of the tissue structure by recording backscattered radiation in both the initial (the first image) and orthogonal polarizations (the second image) and matching pairs of such images. CP OCT is more informative for connective tissue study since the spatial structure of collagen fibers provides not only backscattering of radiation but also a change in its polarization.

For clinical tasks CP OCT is recognized today as the most promising method of carrying out functional diagnostics for reasons of both patient safety and in respect of the simplicity and reliability of the devices used. The use of near-infrared light in these methods as probing radiation is attractive due to its avoidance of causing tissue damage at the powers used and the ability to study deep tissues [13, 14]. In clinical trials, CP OCT has already been applied for the early diagnosis of neoplasia, for identifying boundaries of tumor growth for resection planning, for dynamic monitoring of patients with oncological pathology, and for the timely detection of relapses [15]. CP OCT has also often been used to study the state of the skin [16], mucous membranes when in a healthy state and under various pathological conditions [17], and to monitor the wound healing process in vitro and in vivo [18]. We have not found any studies on skin equivalents using CP OCT; however, there are already several works on the study of skin equivalents using ‘traditional’ OCT [19, 20].

Multiphoton tomography (MPT) is a variant of multiphoton fluorescence microscopy. Clinical MPT provides clinicians and researchers with high-resolution *in vivo* optical biopsies based on two-photon autofluorescence, second harmonic generation, and fluorescence lifetime imaging. MPT with near-infrared femtosecond-pulsed lasers enables *ex vivo* and *in vivo* noninvasive investigation of the skin with subcellular resolution (0.5 μm in the lateral direction and 1–2 μm in the axial direction) [21–25]. Due to naturally occurring, endogenous, fluorescent biomolecules such as flavins, NAD(P)H coenzymes, metal-free porphyrins, lipofuscin components, melanin, elastin, collagen. and keratin, such fluorescence imaging can be performed without additional contrast agents. Second harmonic generation (SHG), caused mainly by fibrillar collagen fibers, is unique to MPT and provides additional information on the tissue content since the autofluorescence spectra of the endogenous fluorophores frequently overlap [26]. Presently, MPT is being successfully applied in clinical dermatology for the study of different dermatoses such as benign and malignant skin cancers, connective tissue diseases, inflammatory skin diseases, and autoimmune bullous skin diseases [27], in the clinical study of healing in chronic wounds (ulcus ulcera) [28], and to provide optical biopsies during resection of a clinical case of glioblastoma [29].

Fluorescence lifetime imaging microscopy (FLIM) is also used in biomedical science as it offers noninvasive, real-time measurements, an optical sectioning capability with high photon efficiency, high lifetime accuracy, and simultaneous recording at several wavelength intervals [30]. FLIM provides more biochemical and medical diagnostic information for the identification of abnormal tissues in vivo than traditional methods based on fluorescence intensity determination, and thus has the potential for wide application in clinical medical diagnostics. Multiphoton excitation FLIM can significantly improve the imaging depth in tissues, by up to 200 in the case of human skin autofluorescence lifetime imaging. Fluorescence lifetime distribution in the tissues can be used to diagnose some skin diseases (cancer, psoriasis, dysplastic nevi) as it can reveal changes in cell metabolism. Noninvasive FLIM, combined with multiphoton tomography (MPT-FLIM), has been used to accurately determine the distribution and metabolic activity of mitochondria during inflammation, and is expected to be valuable in studies of the pathophysiological mechanisms of inflammatory skin diseases and in their early clinical diagnosis and treatment. In addition, MPT-FLIM can accurately identify the boundaries of tumors, informing usefully on the clinical basis for surgical treatment [31].

Clearly, each of these methods provides important data at particular levels—СР OCT at the tissue level, and МРТ and FLIM at the cellular level—but includes information on metabolic activity. In assessing living SEs, both before and after transplantation, it is important to obtain a complete picture regarding the overall structure of the sample, the matrix structure, and the quality of the cells. Therefore, an integrated approach (CP OCT, MPT, and FLIM), in our view, provides an ideal information profile for clinical and research applications.

Therefore, in the current work we report on the investigation of trimodal label-free imaging of the structure and quality of living dermal equivalents. The dermal equivalent structure was examined using CP OCT to determine the collagen structure. The SHG of collagen and the fluorescence lifetimes of NAD(P)H in the cells were traced using MPT combined with FLIM.

## Methods

### Cell cultures

Mouse dermal fibroblasts (FBs) and dermal papilla (DP) cells were used in this study. The DP cells were isolated from the whiskers of C57Bl6 mice using microsurgical manipulation under a binocular microscope [32]. The lower third of a hair follicle containing the dermal papilla was cut off using microsurgical scissors and incubated in a 0.5% solution of collagenase type I for 30 min at 37 °C.

The dermal papillae of the vibrissae were separated from the epithelial parts of the follicles using microneedles and then placed in 35-mm petri dishes and cultured in AmnioMAX medium (Gibco). The cells were cultured with regular medium changes (once every 1 to 2 days) and, upon the formation of a monolayer, the cells were passaged with a ratio of 1:3 or 1:4.

FBs were isolated from the skin of newborn C57Bl6 mice. Using scissors and a scalpel, the skin was separated, and the resulting flap was cut into strips of 3 × 10 mm, and incubated in a 1% dispase solution for 1 h at 37 °C. After that the epidermis was separated from the dermis along the basal plate line using tweezers. The remaining strip of dermis was washed in phosphate-buffered saline (PBS), and then minced into pieces 2–3 mm in size. Disaggregation of the cells was performed in 0.1% collagenase at 37 °C for 2–3 h under visual control. The fermentation was stopped by adding fetal bovine serum (FBS). The resulting cell suspension was pipetted and then centrifuged for 10 min. The supernatant was discarded and the resulting pellet resuspended in culture medium. After the primary cell culture seeding the cells usually formed a confluent layer. At this stage the cells were passaged using a standard procedure, in the ratio 1:2. Subsequently, the cells were cultured with regular changes of medium and, upon reaching monolayer, the cells were passaged with a ratio of 1:3 or 1:4, typically once every 3–4 days.

### Dermal equivalent preparation

Dermal equivalents were constructed by combining the FBs or DP cells with rat tail collagen, type I. The collagen type I solution was prepared by extraction in an acidic solution, a method that provides the most suitable collagen component for preparing collagen gels. The collagen solution was polymerized by changing the pH, with the simultaneous addition of cells and components required to support their viability. To prepare the collagen gel, the following solutions were successively mixed: a sterile 0.34 M solution of NaOH was combined with a concentrated (×10) nutrient medium 199 in a ratio 1:2 and 100 mg of glutamine and 9.5% of sodium bicarbonate were added for every 100 ml of the mixture. The resulting mixture was combined with a cooled solution of collagen in acetic acid in a ratio of 1:4, and then placed on ice to prevent rapid gelatinization. At this stage, a cell suspension was added to the resulting mixture.

This provided a uniform distribution of cells throughout thickness of the gel and also made it possible to obtain gels with the required thickness and diameter. The concentration of collagen was 1.5 mg/ml.

The concentration of cells was chosen according to their contractile ability. It had been shown earlier that cells embedded in collagen gel at concentrations of less than 1 × 10^5^ per ml of gel do not compress it, while at 3 × 10^5^ cells per ml of gel and greater they are able to contract it within 2 days (data not shown). Thus, for the formation of dermal equivalents, 2 × 10^5^ dermal papilla cells (or dermal fibroblasts) per ml of the collagen gel were used as the optimal model for observation over a period of 14 days.

For CP OCT, MPT, and FLIM, the dermal equivalents were transferred into a sterile dish with a cover glass bottom (0.17-mm thick).

### Immunohistochemistry

The contraction abilities of the FBs and DP cells were assessed on the basis of the expression of the specific cytoskeleton proteins vimentin (Abcam, USA) and α-smooth muscle actin (α-SMA; (Abcam, USA) involved in collagen gel contraction.

Proliferating cells were identified by positive staining for Ki67 (Abcam, USA). The presence of specific stem cell markers secreted by the DP cells were assessed by the expression of versican (Abcam, USA) and staining with alkaline phosphatase (Roche Diagnostics, Schweiz), respectively.

The immunohistochemistry and alkaline phosphatase staining was carried out according to standard protocols. The primary antibodies were diluted in the following concentrations: vimentin, Ki67, versican, 1:200; α-SMA, 1:50.

To detect the primary antibodies, secondary antibodies were used conjugated with Alexa-488 or Alexa-546 (dilution 1:200). The nuclei were counterstained with 0.001% DAPI (Santa Cruz, Texas, USA). The stained dermal equivalents were examined using a fluorescence microscope for the purposes of the immunohistochemistry. The immunohistochemical analysis of the prepared dermal equivalents was carried out on days 1, 7, and 14 of cultivation.

### CP OCT

The ability of DP cells and FBs to contract the three-dimensional (3D) collagen gels was analyzed using OCT for the study of the remodeling of the extracellular matrix. In this study, a new spectral domain OCT setup developed by the Institute of Applied Physics was used [33]. This OCT device is characterized by an acquisition rate of 20,000 spectral A-scans per second, a central wavelength of 1300 nm, lateral resolution 15 μm, depth of visualization 1.7 mm, and imaging size 4 × 4 mm. The B-scans and 3D data sets were obtained in a noncontact manner. CP OCT forms two images simultaneously by the separate registration of the scattered radiation in the two channels, parallel and perpendicular to the polarization of the probe radiation, and gives a detailed representation not only in respect of the internal structure but also, most importantly, the functional state of the collagen structures in the tissue.

The dermal equivalents were imaged on days 0, 7, 10, and 14 after their preparation. Five areas were investigated in direct and orthogonal polarization from each sample.

### MPT and FLIM

For more detailed information, the changes in the collagen structure and the quality of the cell components were assessed with the use of MPT and FLIM. The two-photon excited fluorescence intensity and FLIM images were obtained using a multiphoton tomography MPTflex (JenLab GmbH, Germany) equipped with a tunable 80 MHz, 200 fs Ti:Sa laser MaiTai, and a TCSPC-based FLIM module (Becker & Hickl GmbH, Germany). The images were acquired through a 40×, 1.3 NA, oil immersion objective.

NAD(P)H fluorescence was excited at a wavelength of 740 nm and detected in the range 410–650 nm. The average power applied to the sample was ~ 12 mW.

Second harmonic generation (SHG) was excited at 740 nm and detected in the range 373–387 nm to identify oriented collagen fibers in the dermal equivalent. Quantitative analysis of the SHG signal intensity distribution for the collagen was performed using first and second order statistical methods (Integration Density, Energy and Coherence) in ImageJ (National Institutes of Health, USA). For quantitative analysis of the SHG signal the intensity distribution for the collagen at each time point in the dermal equivalents under study (from 5 to 18 regions of interest (ROIs)) was inspected. Higher integration density, coherence, and energy values correspond to structures that are less isotropic and more oriented.

#### Fluorescence lifetime analysis

The fluorescence lifetimes and their contributions (free and protein-bound forms of NAD(P)H: (t1), free NAD(P)H; (t2), bound NAD(P)H; (a1), free NAD(P)H; (a2), bound NAD(P)H) for the ROIs were calculated by finding the global minimum of the χ^2^ value. The mean values of χ^2^ and the fluorescence lifetimes in the FBs and DP cells were assessed in the cell cytoplasm. Bi-exponential fitting was used for the analysis of both cofactors. The FLIM images were processed in SPCImage software (Becker&Hickl GmbH, Germany). For fluorescence lifetime analysis at each time point of the dermal equivalents study, from 5 to 10 randomly selected cells and from 10 to 20 ROIs were inspected.

### Statistical analysis

Statistical analysis was performed using STATISTICA 64 software, version 10 (StatSoft Inc., Tulsa, OK, USA). Mean and standard deviation (SD) values were used to express the data. Differences in the mean values were tested for significance using the Student’s *t* test or one-way analysis of variance (ANOVA) with Fisher’s post hoc test (*p* ≤ 0.05).

## Results

### Characterization of dermal equivalents

Both the DP cells and FBs stretched, formed multiple sproutings, and started to contract the collagen gel during the first day after being embedded in it.

**Fig. 1:**
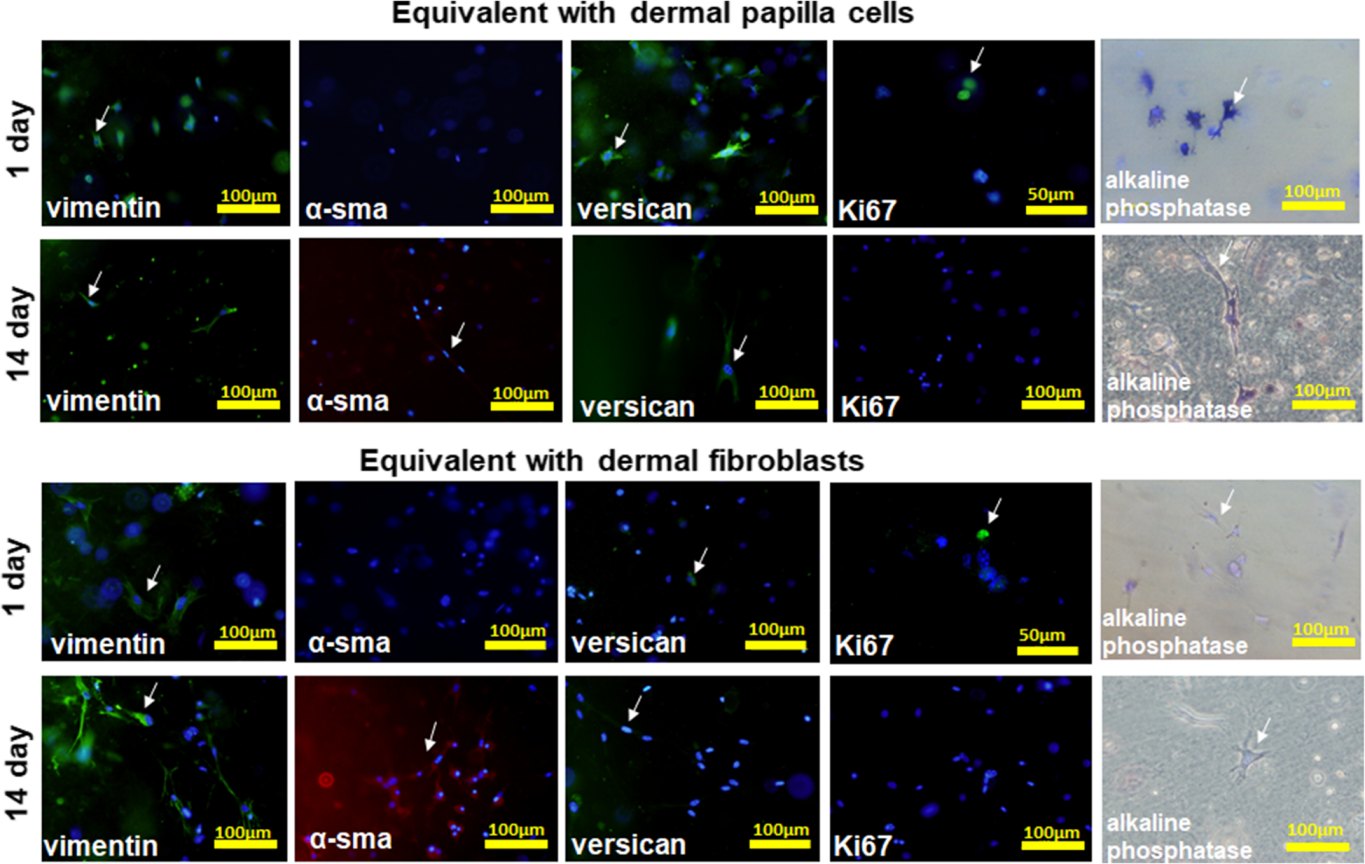
Immunohistochemical analysis of dermal equivalents with dermal papilla cells and with dermal fibroblasts at days 1 and 14 of cultivation. Immunofluorescence detection: vimentin (green staining), α-smooth muscle actin (α-sma; red staining), versican (green staining), Ki67 (green staining), and alkaline phosphatase (dark staining). Nuclei were counterstained with DAPI (blue staining). Scale bars = 50 μm and 100 μm. Cells are marked by arrows

Throughout the culturing of the dermal equivalents the cells expressed their specific markers. Vimentin was expressed in both the DP cells and FBs for the entire culture period (14 days). α-SMA was detected in both cell types only on the final day (14) of cultivation. However, proliferating cells (positively stained for Ki67) were found only on the first day of cultivation. Versican (a specific protein secreted by DP cells) at the confidence level was detected in the DP cells at all stages (Fig. 1). Furthermore, weak expression of this marker was evident in the FBs by 2 weeks of cultivation. This can be explained by the presence in the FB culture of a small number of DP cells retained after isolation from the skin. Alkaline phosphatase (a stem cell marker) was reliably revealed in the DP cells at all stages. On day 14 of cultivation, alkaline phosphatase staining was also found in some cells in the FB cultures (Fig. 1). This fact is probably due to the appearance of poorly differentiated cells in the FB culture after prolonged cultivation.

### Assessment of the structure of dermal equivalents using CP OCT

For a general study of extracellular matrix remodeling we used CP OCT to analyze the ability of DP cells and FBs to contract 3D collagen gels *in vitro*.

**Fig. 2:**
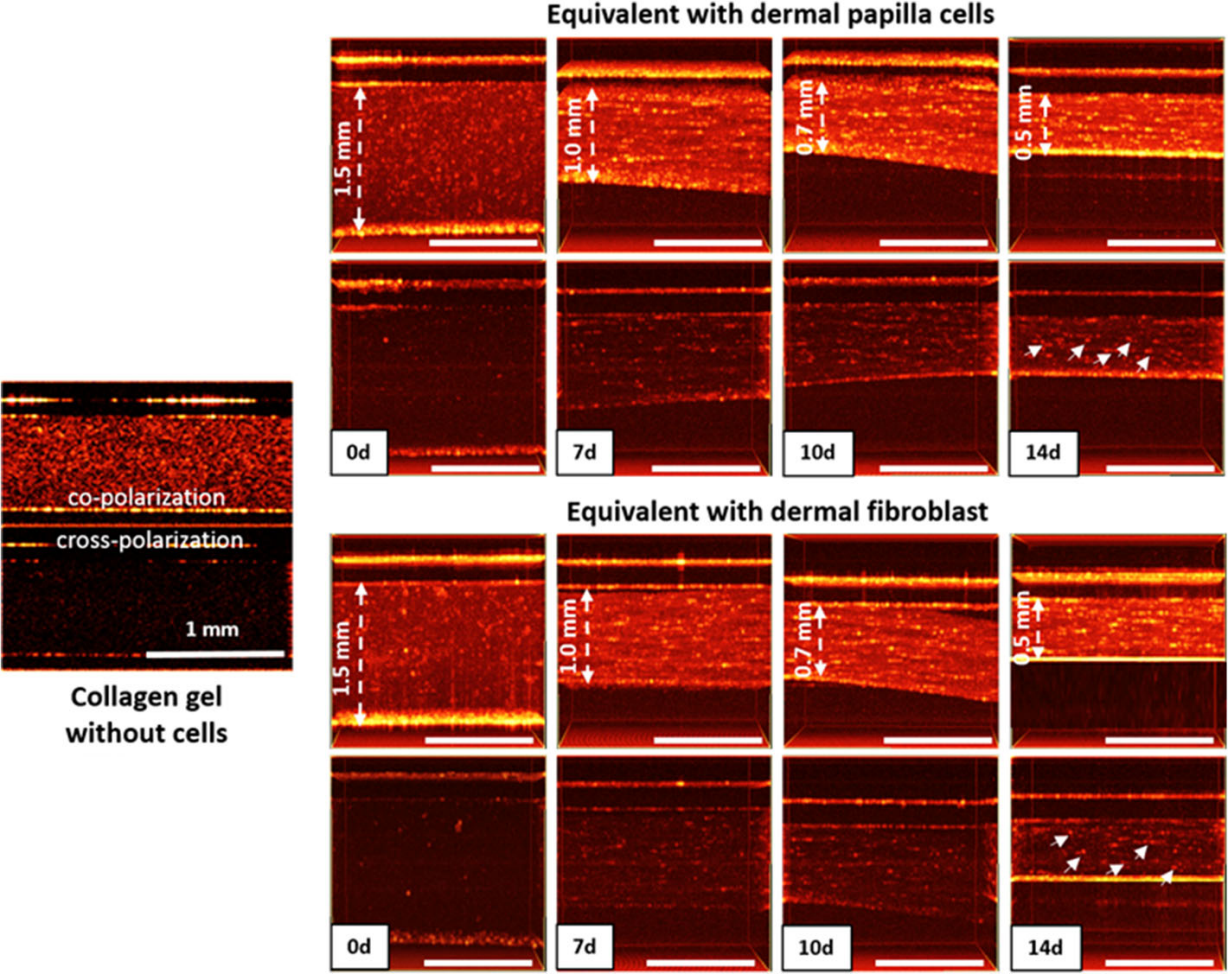
CP OCT monitoring of dermal equivalents with DP cells or with FBs on days 0, 7, 10, and 14 after formation of the equivalents. Dashed arrows indicate the thickness of the dermal equivalents. Areas with high CP OCT signals are marked by solid arrows. Scale bars = 1 mm

In preliminary experiments, the collagen gel without cells was evaluated. It was found that it was characterized by a high and uniform OCT signal in co-polarization and by the lack of an OCT signal in cross-polarization (Fig. 2).

Monitoring of dermal equivalent formation was performed by CP OCT. Immediately after preparation, the OCT images of the dermal equivalents were similar to the OCT images of the collagen gel; they were characterized by a high-intensity homogeneous OCT signal on the co-polarization images and the complete absence of an OCT signal on the cross-polarization images (Fig. 2). The thickness of both equivalents was approximately the same, at about 1.5 mm.

Further observation (day 7) revealed a significant decrease in the thickness of the dermal equivalents (reduced to 1 mm). At the same time we observed an increase in the OCT signal in co-polarization and the appearance of a low OCT signal in the cross-channel. Subsequently, the thickness of both equivalents reduced to 0.7 mm (day 10). In cross-polarization the OCT signal rose, indicating the formation of anisotropic structures possessing polarizing properties (for example, collagen fibers). On day 14, the thickness of the samples, maximally, had decreased (to 0.5 mm). No significant differences in the character of the OCT signal in co- and cross-polarization in either of the dermal equivalents were detected (Fig. 2).

### Assessment of the structure and quality of dermal equivalents using MPT and FLIM

For more detailed information, changes in the collagen structure and the quality of the cell components were assessed with the use of MPT combined with FLIM.

**Fig. 3:**
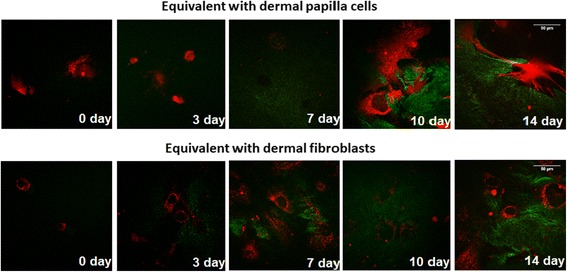
Superimposed two-photon excited autofluorescence of cells (red), and the SHG of collagen (green) images of dermal equivalents with DPs or with FBs on days 0, 3, 7, 10 and 14 after equivalent formation. The image size is 205 × 205 μm (1024 × 1024 pixels). Scale bars 50 μm

The visual assessment of the collagen structure showed an elongation of the DP cells and FBs in the collagen gel during the early stages of dermal equivalent formation (day 0). On day 3, all viable cells were fibroblast-like in shape. A low-intensity SHG signal was found in the dermal equivalent with fibroblasts and the presence of dermal papillae suggested the beginning of collagen fibril formation. From days 3 to 10, no further significant changes were detected in either of the dermal equivalent types. A significant SHG signal increase was revealed in both the dermal equivalents on day 10. The maximum rise of the SHG signal was also detected in both the dermal equivalents on days 12 and 14 (Fig. 3).

To quantitatively evaluate the presence, local organization, and isotropic properties of the collagen fibrils, the integration density, energy, and coherence were selected as the output parameters.

**Fig. 4:**
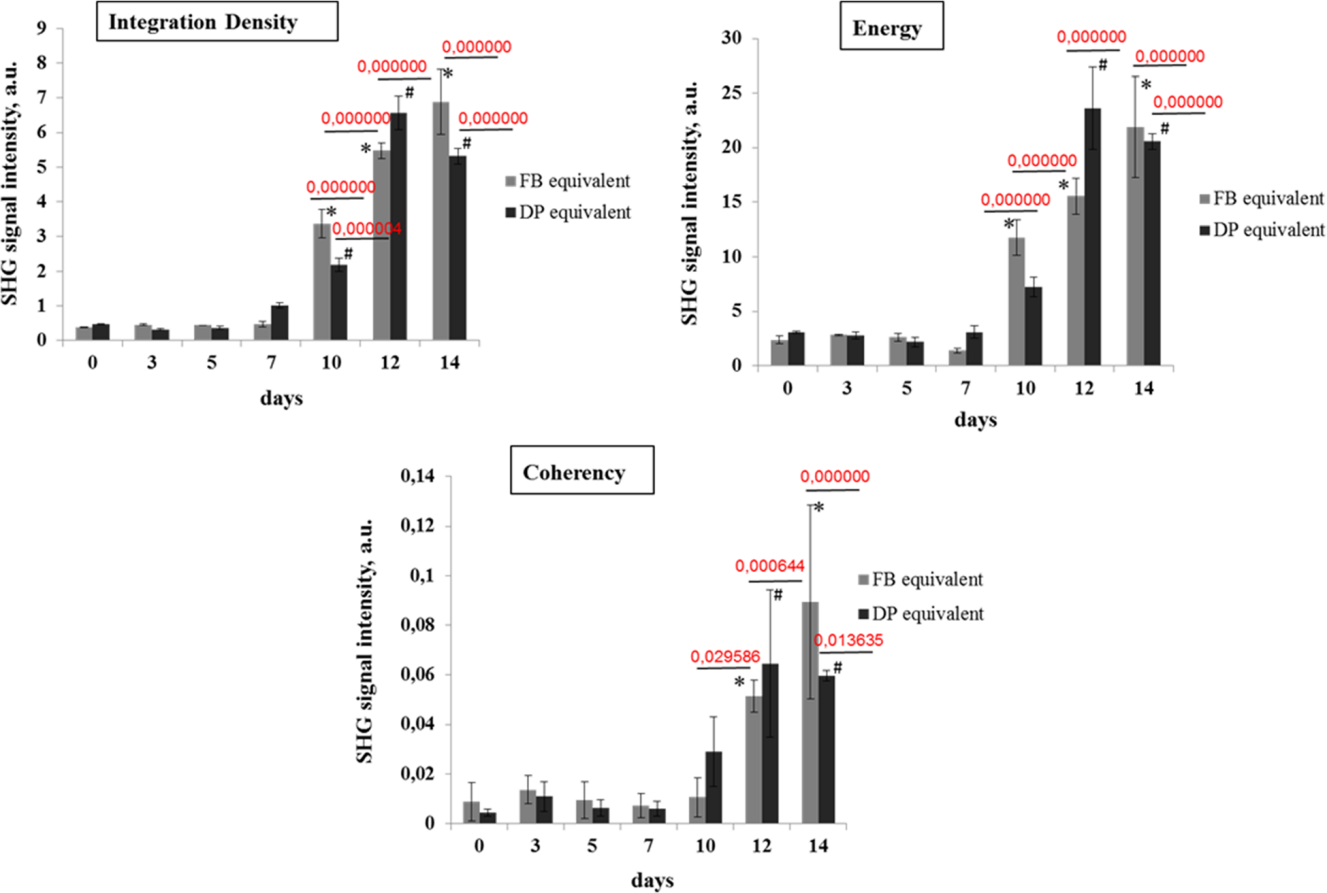
Dynamics of the values of integration density, energy and coherence of collagen in dermal equivalents with dermal papilla (DP) cells or with dermal fibroblasts (FBs). Mean ± SD. *Statistically significant differences in values in dermal equivalents including FBs compared with the corresponding values on day 0; ^#^statistically significant differences in values in dermal equivalents including DP cells compared with day 0. SHG, second harmonic generation

In the dermal equivalents with DP cells, a dynamic increase in these parameters occurred, but only until day 12. On day 14 the values of integration density, coherence and energy had decreased compared with their values on day 12. In the case of the dermal equivalents with FBs, corresponding dynamic changes in the values of the selected parameters were found at all time points. However statistically significant increases in these parameters were detected only on days 10, 12 and 14 (integration density and energy) and on days 12 and 14 (coherence) (Fig. 4).

It should be noted that the equivalents with DP cells had lower values of integration density (2.18 ± 0.19 vs 3.36 ± 0.41, *p ≤ 0.000794*) and energy (7.24 ± 0.88 vs 11.74 ± 1.61*, p ≤ 0.000029*) on day 10 compared with the equivalents with FBs. However, the values of integration density (6.57 ± 0.49 vs 5.48 ± 0.23, *p ≤ 0.001907*) and energy (23.6 ± 3.78 vs 15.56 ± 1.64, *p ≤ 0.002559*) had increased on day 12 but had fallen again (5.31 ± 0.22 vs 6.88 ± 0.93, *p* ≤ 0.018628 and 20.59 ± 0.72 vs 21.91 ± 4.62, *p* ≤ 0.642676) by day 14.

There were no statistically significant differences between the coherence values for the different types of dermal equivalents. The decrease in integration density and energy values on day 14 was probably due to behavioral changes of the DP cells once they had achieved maximum contraction. Also, the ability of the DP cells to digest the matrix by means of extensive metalloprotease secretion by day 14 cannot be excluded [34].

Therefore, along with the CP OCT data, these results suggest the same manner of collagen gel structuring in both types of dermal equivalent.

### FLIM analysis

For the assessment of cell component quality, we analyzed the fluorescence lifetimes and lifetime contributions of the free and protein-bound forms of NAD(P)H as indicators of the energy metabolism in the FBs and DP cells. It is known that a bias toward oxidative metabolism results in a higher lifetime value and a higher contribution from protein-bound NAD(P)H [35].

**Fig. 5:**
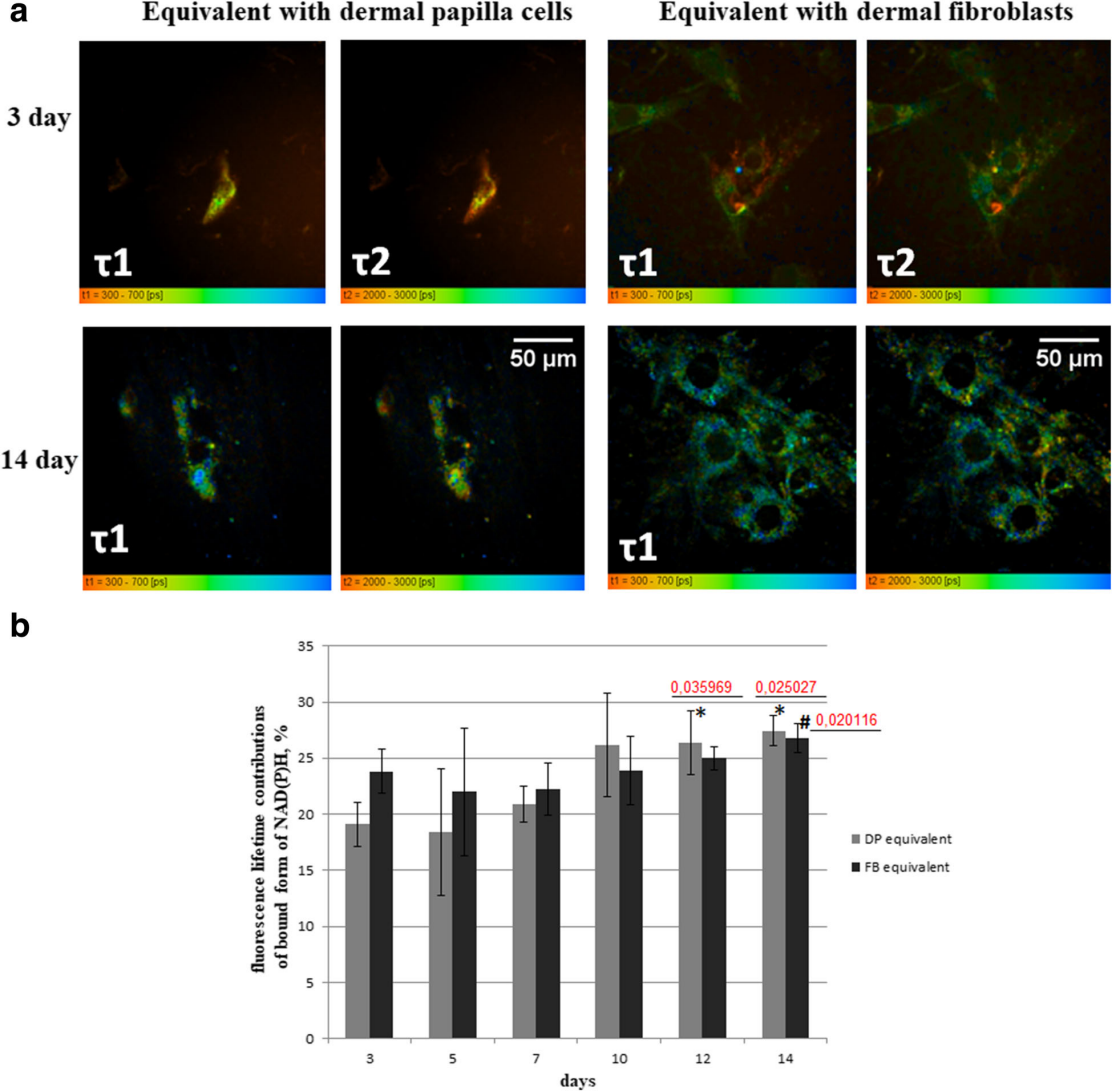
FLIM of NAD(P)H in dermal papilla (DP) cells and dermal fibroblasts (FBs) included in dermal equivalents. **a** Pseudocolor-coded images of the free (t1) and protein-bound (t2) forms of NAD(P)H. Field of view 205 × 205 μm (127 × 127 pixels). **b** Dynamics of the fluorescence lifetime contributions of the protein-bound forms. Mean ± SD. *Statistically significant difference for NAD(P)H in dermal equivalent including DP cells on day 3; ^#^statistically significant difference for NAD(P)H in dermal equivalent including FBs on day 3. *P* values are shown. Scale bars = 50 μm

On day 14, we found that for the equivalents with DP cells the fluorescence lifetimes of the free (t1) and bound (t2) forms of NAD(P)H were higher than in the cells on day 3. However, a statistically significant increase on day 14 was revealed only in the short lifetimes of NAD(P)H (Fig. 5a).

The fluorescence lifetime contribution of protein-bound NAD(P)H (a2) in the DP cells on days 12 and 14 was higher than in the cells on day 3, in general indicating a more oxidative state (Fig. 5b).

In the case of the dermal equivalents with FBs, the fluorescence lifetimes of the free (t1) and bound (t2) forms of NAD(P)H showed statistically significant increases on days 10 and 14 compared with that of the cells on day 3 (Fig. 5a). The contribution of the protein bound NAD(P)H (a2) in the FBs was higher only on day 14 when compared with the dermal equivalent on day 3 (Fig. 5b).

DP cells in collagen gel on day 3 showed lower values of the contribution of bound NAD(P)H (a2) (*p* ≤ 0.000264) as compared with the FBs. However, after day 10 the contribution of bound NAD(P)H in the DP cells increased and showed higher values on day 14 compared with the FBs (although this was not a statistically significant difference).

Therefore, these results suggest a shift to a more oxidative metabolism in dermal papilla cells and dermal fibroblasts on day 14 when cultured in three-dimensional collagen gel.

## Discussion

Тrimodal label-free imaging is a powerful tool for preclinical and clinical skin equivalent estimation. In this study, we simultaneously applied several visualization techniques for skin model exploration. We investigated the structure of dermal equivalents with DP cells and FBs using CP OCT and MPT (SHG) and their quality using the FLIM method. Both the structuring of the collagen fibrils and the energy metabolism of the cellular components were addressed.

In the current study of extracellular matrix remodeling, we used CP OCT and MPT to analyze the ability of DP cells to contract three-dimensional collagen gels *in vitro*. Currently, extracellular matrix remodeling by DP cells has been very poorly investigated. Moreover, it was thought that the contraction ability of DP cells when compared with FBs displayed opposite trends. It was also demonstrated that FBs and DP cells are very comparable in their contraction ability, and DP cells show the same fractional forces as FBs. Immunohistochemical analyses indicate the presence of the corresponding cytoskeletal proteins, vimentin, tubulin, and actin in both types of cells [36]. Hill et al. have shown that both dermal sheath (DS) and DP cells seeded into rat tail type I collagen contracted the collagen lattice to a similar degree, with both displaying significantly enhanced contractile ability over dermal FBs. DP cells and DS cells expressed more α-SMA and were more contractile than dermal FBs [37]. However Almond-Roesler et al. revealed a significantly lower capacity of DP cells to reorganize extracellular matrix components than that of dermal FBs and, moreover, the DP cells lysed the collagen lattices completely after 48 h in culture [38]. In our work, the gel contraction was very similar for both dermal FBs and DP cells, showing that DP cells can cause the same manner of collagen gel structuring as FBs. In both types of cell, visible collagen fibril formation started at 3–7 days with the maximum elevation of the CP OCT and SHG signals being registered from day 10. Moreover, in both cases, between days 12 and 14, we detected the type of higher coherence and energy values associated with the formation of structures that were less isotropic and showed greater orientation. This ability coincides with the immunohistochemical demonstration of the presence of the same complement of the cytoskeletal element vimentin in both dermal papilla cells and fibroblasts.

However, it should be noted that we observed negligible differences in the contraction ability of DP cells on day 14 compared with the contraction ability of collagen on day 0. DP cells on day 14 displayed reduced values of integration density, energy, and coherence and this was probably due to behavioral changes of the DP cells once they had achieved maximum contraction. It is known that DP cells secrete the extensive metalloprotease and this means that their ability to digest the matrix by day 14 cannot be excluded [34].

Besides the study of the ability of cell components for matrix remodeling, it is important to know the metabolic activity of the cells included in the skin equivalents. The metabolic status of the cells provides information not only about cell metabolic and synthetic activity but also about their possible differentiation [39]. Our work based on the fluorescence lifetimes and contributions of protein-bound NAD(P)H indicated a bias toward oxidative metabolism in DP cells and in FBs on day 14 of cultivation. To the best of our knowledge, FLIM has never previously been applied to the study of metabolic changes in DP cells and FBs included in dermal equivalents.

It is known that DP cells are related to MSCs. Recent studies have shown that in highly proliferating stem cells which have increased needs for energy (ATP) glycolysis predominates, while differentiated cells typically produce ATP through oxidative phosphorylation [35, 39, 40]. The bias of DP cells towards an oxidative status may be related to their higher energy needs due to their contraction of the collagen gel. However, the data concerning their decrease in proliferative activity suggest that possible differentiation of the DP cells cannot be excluded [41, 42].

In the case of FBs we also observed a bias toward oxidative metabolism, but this was more evident in the late stages (day 14). It is known that skin fibroblasts normally use a highly glycolytic mode of metabolism. However, this mode can become more oxidative due to decreased cell growth and ATP turnover, creating less demand for ATP synthesis by glycolysis [43]. Indeed, proliferating FBs, positively stained for Ki67, were found only on day 1 of culture. Therefore, we suggest that the FBs in living dermal equivalents had a more oxidative status due to their decreased cell growth and the greater energy needs for contracting the collagen gel.

Our work represents the first use of the combination of these visualization techniques in skin equivalent research. However, it is important to note that there are a small number of works where other researchers have also used a combination of different methods for SE imaging. For instance, Pena et al. demonstrated the usefulness of multiphoton microscopy (MPM) in visualizing the three-dimensional remodeling of the extracellular matrix induced by dermal fibroblast contraction, or by other processes, in unstained engineered dermal tissue [44]. Urciuolo et al. used multi-photon microscopy to investigate a three-dimensional human dermis equivalent (3D-HDE) as an *in vitro* model to study collagen network rearrangement under simulated solar exposure. SHG and two-photon excited fluorescence (TPEF) imaging have also been used to assess modifications in collagen assembly before and after UV irradiation [45]. Thus, Barton et al. studied the structure of three-layer skin-equivalent models (rafts) at 10 days after their construction using a trimodality microscope including optical coherence (OC), two-photon excited fluorescence (TPEF), and second harmonic generation (SHG) channels. Their results indicated strong SHG from the collagen in the collagen/fibroblast layer (dermis) with hypointense regions that may correspond to the locations of the cells. The OC signal had a characteristic texture, and also showed hypointense regions in the same locations as did the SHG channel [46].

**Fig. 6:**
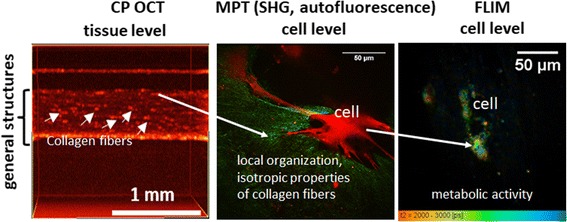
Potential of an integrated approach using cross-polarization optical coherence tomography (CP OCT), multiphoton tomography (MPT), and fluorescence lifetime imaging microscopy (FLIM) in skin equivalent preclinical research. SHG, second harmonic generation

Given the above, we speculate that, compared to other works, our trimodal approach should be able to provide more complete data acquisition on the structure and quality of SEs at all levels of preclinical research: 1) the OCT technique can visualize the general structures of SEs, the presence of collagen fibers in them, and provide data regarding extracellular matrix remodeling at a tissue level; 2) MPT can give more accurate information about the collagen structures (presence, local organization, and isotropic properties) using the SHG mode and the presence of cell components using the autofluorescence mode at the cell level; and 3) FLIM can characterize more precisely the cell quality (metabolic activity) at a cellular level (Fig. 6).

We also suggest that the combination of CP OCT, MPT, and FLIM has the potential to be applied in the clinic for the investigation of skin equivalents before and after transplantation for wound healing. The results of our study provide a basis for the label-free monitoring of: 1) possible stem cell differentiation in skin equivalents with the use of two-photon FLIM for determining the metabolic status; and 2) collagen fiber remodeling and derma formation using МPT (SHG) and CP OCT.

## Conclusion

In this study, trimodal label-free imaging of the structure and quality of living dermal equivalents was implemented for the first time with the use CP OCT, MPT (SHG), and FLIM of NAD(P)H. Our data suggest that the combination of different imaging techniques provides an integrated approach to data acquisition on the structure and quality of such dermal equivalents, minimizes the potential disadvantages of using a single method, and provides an ideal information profile for clinical and research applications.

## Abbreviations

ATP: Adenosine triphosphate
CP OCT: Cross-polarization optical coherence tomography
DP: Dermal papilla
DS: Dermal sheath
FB: Dermal fibroblast
FLIM: Fluorescence lifetime imaging microscopy
MPT: Multiphoton tomography
MSC: Mesenchymal stromal cell
NAD(P)H: Nicotinamide adenine dinucleotide (phosphate)
OCT: Optical coherence tomography
ROIs: Regions of interest
SC: Stem cell
SE: Skin equivalent
SHG: Second harmonic generation
SMA: Smooth muscle actin
